# Identification and dynamics of a beneficial mutation in a long-term evolution experiment with *Escherichia coli*

**DOI:** 10.1186/1471-2148-9-302

**Published:** 2009-12-29

**Authors:** Mark T Stanek, Tim F Cooper, Richard E Lenski

**Affiliations:** 1 Department of Microbiology and Molecular Genetics, Michigan State University, East Lansing, Michigan 48824-4320, USA; 2 Department of Biology and Biochemistry, University of Houston, Houston, Texas 77204, USA

## Abstract

**Background:**

Twelve populations of *E. coli *were serially propagated for 20,000 generations in a glucose-supplemented minimal medium in order to study the dynamics of evolution. We sought to find and characterize one of the beneficial mutations responsible for the adaptation and other phenotypic changes, including increased cell size, in one of these populations.

**Results:**

We used transposon-tagging followed by P1-transduction into the ancestor, screening for increased cell size and fitness, co-transduction analysis, and DNA sequencing. We identified a 1-bp insertion in the BoxG1 region located upstream of *glmUS*, an operon involved in cell-wall biosynthesis. When transduced into the ancestor, this mutation increased competitive fitness by about 5%. This mutation spread through its population of origin between 500 and 1500 generations. Mutations in this region were not found in the other 11 evolving populations, even after 20,000 generations.

**Conclusion:**

The 1-bp insertion in the BoxG1 region near *glmUS *was demonstrably beneficial in the environment in which it arose. The absence of similar mutations in the other evolved populations suggests that they substituted other mutations that rendered this particular mutation unimportant. These results show the unpredictability of adaptive evolution, whereas parallel substitutions at other loci in these same populations reveal the predictability.

## Background

Evolutionary change involves a fundamental tension between chance and necessity [1]. On the one hand, mutations that produce heritable variation occur at random, although rates may be modulated by genetic and environmental factors [2]. On the other hand, natural selection tends systematically to increase the frequency of those mutants with phenotypic properties that are useful in a particular environment. How these forces play out in time has been the subject of important speculation [3,4] but is difficult to address empirically.

One approach that can rigorously address the interplay between chance and necessity is experimental evolution, especially using microorganisms. In particular, one can examine the evolution of populations founded from the same ancestral genotype and propagated under identical environmental conditions. Unlike experiments with plants and animals, an evolving population can be founded from a single haploid individual, such that there is no shared genetic variation (identical by descent) that would tend to exaggerate parallel changes. Several recent studies with viruses and bacteria have demonstrated striking examples of parallel genetic changes, affecting the same loci and sometimes even the same base-pair, that evidently confer fitness benefits [5-15]. Some of these studies have also found mutations that are present in only one of the replicate populations, but without construction and analysis of isogenic clones it is unclear whether these unique mutations are beneficial or, alternatively, are neutral or even deleterious mutations that hitchhiked with beneficial mutations.

In the longest running evolution experiment with microorganisms, 12 populations of *Escherichia coli *B have been serially propagated in a glucose-supplemented minimal medium for tens of thousands of generations [16-18]. During this time, the average competitive fitness increased by ~75% relative to the ancestor [18], the average cell volume substantially increased [17-21], and many other phenotypic changes occurred [11,12,18,22,23]. A number of mutations have been found in these populations by RFLP screening using IS elements as probes [24,25] as well as by random, targeted, and whole-genome sequencing [11,12,21-23,26-28]. Some of these mutations have been demonstrated to be beneficial in the experimental environment by constructing and competing clones that differ only by a single mutation [reviewed in 28]. In most cases, those genes bearing mutations that were demonstrated to be beneficial also harboured mutations in multiple replicate populations. These parallel mutations affected the same genes, but they were not usually identical at the sequence level.

The primary goals of the present paper are to identify a beneficial mutation based on its advantageous effect; and then to determine whether the same or similar mutations were substituted in the replicate evolved populations. Our strategy was as follows. Using an evolved clone, we made a pool of clones each carrying a randomly inserted marker. We then used this entire pool as donors, and transduced the marker and linked genes into the ancestor. Next, the resulting transductants were tested for increased cell size and competitive fitness. A clone with both properties served as a donor for a second round of transduction, in order to estimate the physical distance between its marker and the beneficial mutation of interest. We then sequenced the relevant regions to find any mutations. We found two mutations, one of which was previously unknown. We also sequenced the same region in clones from samples that were stored at various times to characterize the dynamics of this mutation in the focal population. Finally, we sequenced this region in clones from the replicate populations to determine whether the same or similar mutations had been substituted in them.

## Results

### Finding a transduced clone that carries a beneficial mutation

REL4548 is a clone that was isolated after evolving for 10,000 generations in a constant environment. From REL4548, we produced a mix of 1296 insertion mutants, each carrying a Tn*10 *mini-transposon that confers resistance to tetracycline. A P1 lysate was then made from that mixture of insertion mutants, and the lysate was used to transduce the ancestral strain, REL606 (see Methods for details). The total pool of transductants was propagated for 7 days (~47 generations) in the same environment in which the long-term evolution experiment was conducted. This procedure should increase the frequency of beneficial transductants that were initially present in the pool, but the period is too short to allow significant de novo evolution. In the long-term experiment itself, the first increases in fitness were discerned around generation 200, and their magnitudes were consistent with mutations having about a 10% fitness advantage [16,17]. The 7-day enrichment of a mutation that confers a 10% advantage should increase its relative frequency by about 25-fold, and smaller effect mutations would be enriched to a lesser extent. Therefore, this approach is more likely to find beneficial mutations with large effects than those with small effects. To identify beneficial transductants we first screened for larger cell size, which has been shown to be strongly correlated with higher fitness in that environment [17-19]. Measurements of cell size are easier than measurements of relative fitness, which means that more transductants can be screened for the former than for the latter. Those transductants with increased average cell size were then subsequently tested for improved fitness.

The average cell volume of the ancestral clone at stationary phase was measured as between 0.37 and 0.39 fl. Among the enriched transductant pool, there was a second mode in the cell-volume distribution centred around 0.43 fl. We initially chose ten clones that had average cell volumes of ≥0.44 fl, and we measured the competitive fitness of each relative to the ancestor. Of these, three clones were then chosen for further study because each one exhibited a statistically significant increase in fitness. Southern hybridization analyses further showed that these three candidates had the same transposon insertion and hence were progeny of the same transduced clone. One of them, designated REL10247, was chosen for further characterization.

### Co-transduction analysis of the transposon and the beneficial mutation

Our next challenge was to locate the beneficial mutation linked to the mini-transposon insertion. P1-transduction can move as much as ~2% of the bacterial chromosome; thus, the beneficial mutation could lie as far away as 100,000 bp from the inserted transposon. To get some idea of the distance between the transposon-encoded Tet^r ^marker and the beneficial mutation, we examined the co-transduction of the resistance marker and increased cell size, using REL10247 as the donor and the ancestor as the recipient.

The average cell volumes were measured for over 300 Tet^r ^transductants, and 91.5% produced large cell sizes consistent with co-transduction. Based on the relationship between co-transduction frequency and physical distance [29], using 9.66 × 10^4 ^bp as the length of the transducing DNA [29] and 4.6 × 10^6 ^bp for the genome size [30], this frequency predicts a distance of about 2800 bp between the beneficial mutation and the inserted transposon.

### Identification and fitness effect of the beneficial mutation

The second piece of information necessary to locate the beneficial mutation was to map the inserted mini-transposon. Genomic DNA from the REL10247 clone was digested with *Ava*I and shotgun-cloned into pUC19. A Tet^r ^colony was chosen, the plasmid isolated, and the inserted fragment was sequenced with standard M13/pUC forward and reverse sequencing primers. The sequence corresponded to the 3' end of the *glmUS *operon. Another primer was then used to sequence the junction between the mini-transposon and the *E. coli *genome. These data showed that the transposon had inserted just beyond the *glmUS *operon, 20 bp downstream from the final amino acid of the GlmS protein.

**Figure 1:**
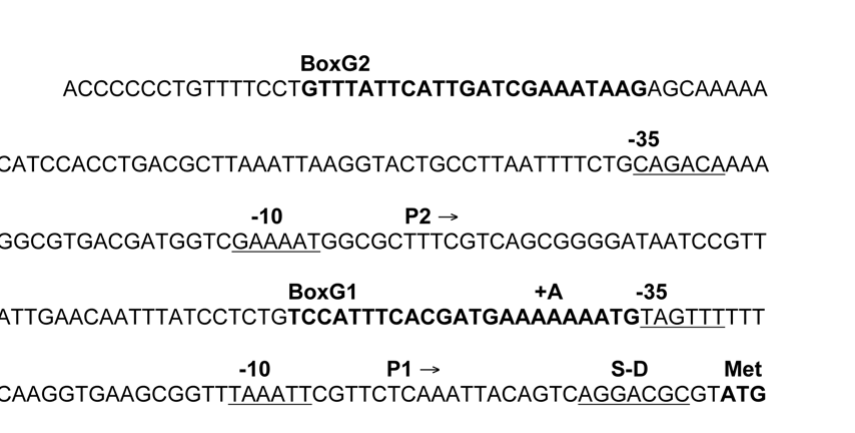
**Region upstream of *glmUS*, including site of beneficial mutation**. NagC binds to two 23-bp BoxG elements shown in bold. The beneficial adenine insertion was in a tract of seven existing adenines in BoxG1; it is shown above the sequence as +A. The Shine-Dalgarno sequence (S-D) and the -10 and -35 regions of two promoters, P1 and P2, are underlined. Figure modified from Plumbridge [31].

**Figure 2:**
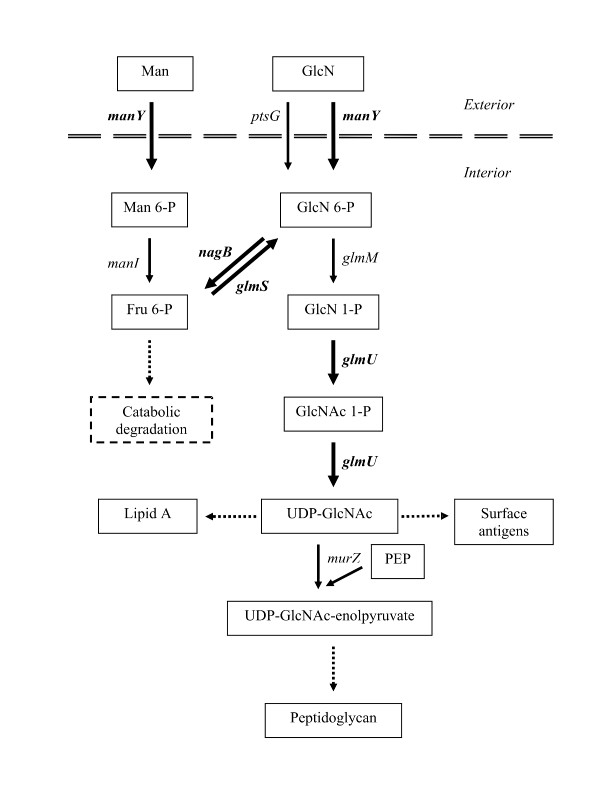
**Biochemical pathways involving GlmS and GlmU**. Biochemical pathways involving GlmS and GlmU, along with certain transport and biosynthetic reactions. Not all participating molecules are shown for every reaction. Genes encoding relevant enzymes are indicated next to the reactions. Genes printed in bold, and next to reactions shown as heavier arrows, are regulated by NagC. The dashed horizontal line represents the cell envelope. Dashed arrows indicate multiple steps. This figure is a composite of information from several sources [31-33,41,59].

Four sets of partially overlapping primer pairs were designed to amplify ~3000 bp of DNA each. Using these primers, DNA was sequenced from both the ancestor and the 10,000-generation clone from which REL10247 was derived, and new primers were synthesized using the 3' end of the sequence to walk along each fragment. Using this approach, we sequenced ~12,000 bp total, about half on each side of the transposon insertion. Only one difference between the ancestor and evolved clone was found in this entire region, a 1-bp adenine insertion in a homopolymeric tract of seven existing adenines. This tract is in a NagC protein-binding site, called BoxG1, near the *glmUS *P1 promoter [31] (Figure 1). The *glmUS *operon encodes two proteins: GlmS converts fructose-6-phosphate (Fru6P) to glucosamine-6-phosphate (GlcN6P); GlmM (not part of the operon) converts GlcN6P to glucosamine-1-phosphate (GlcN1P); and GlmU, a bi-functional enzyme, converts GlcN1P via two reactions to uracil-diphosphate-N-acetyl-glucosamine (UDP-GlcNAc), a precursor in the syntheses of both peptidoglycan and lipopolysaccharides [32,33] (Figure 2). The 1-bp insertion mutation is 3443 bp from the inserted mini-transposon, which agrees well with the 2.8 Kb predicted by the co-transduction analysis. We refer henceforth to this mutation as BoxG1^8A ^in recognition of the insertion of an eighth adenine in the tract.

To demonstrate conclusively that the BoxG1^8A ^mutation in *glmUS *was responsible for the fitness increase of the REL10247 strain, we moved that mutation into the ancestor using the gene gorging method [34] to make strain TC640. This strain had a fitness of 1.05 ± 0.01 (95% confidence interval) relative to the ancestor. This advantage was somewhat less than measured for strain REL10247 itself, suggesting that REL10247 contains one or more additional beneficial mutations. By screening REL10247 for known beneficial mutations in the vicinity of *glmUS*, we found that it also has a deletion of the majority of the *rbs *operon, which was previously shown to confer a fitness benefit of ~1.5% [11].

**Figure 3:**
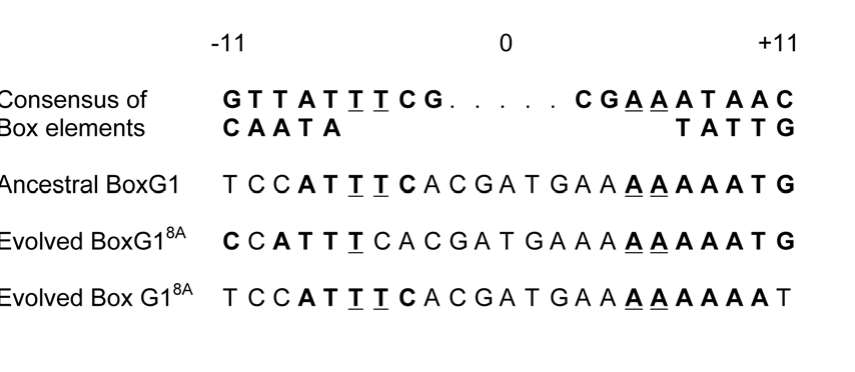
**Effect of BoxG1^8A ^mutation on NagC binding sequence**. The consensus sequence, including alternative bases at some sites, for a 23-bp NagC-binding Box element is shown at the top [31]. The four underlined bases are the only ones that are invariant across all such elements [31], and mutagenesis has shown that these four are also the most important for binding NagC [38]. The next row shows the ancestral BoxG1 sequence. The bottom two rows show the effect of inserting an extra adenine, as in the evolved BoxG1^8A ^allele, when viewed as shifting the sequence either to the left or to the right. The former eliminates the invariant T at position -5. The latter removes agreement with the consensus at position +11.

The beneficial BoxG1^8A ^mutation presumably alters binding of the transcription factor NagC to the BoxG1 region. The NagC protein acts as a transcriptional repressor for the *nagE-BACD *and *manXYZ *operons, but as a transcriptional activator for the *glmUS *genes [31,35,36]. NagC binds to elements associated with two promoters of the *glmUS *operon: BoxG1 located next to the proximal promoter P1, and BoxG2 located upstream of the distal promoter P2 [31]. P1 activity is stimulated by growth on glucose, whereas P2 activity is fairly constant across a range of conditions. NagC binding is required for activation of the P1 promoter [31]. NagC binds to a 23-bp consensus sequence [31,35,37-39] (Figure 3). Mutagenesis studies have shown that the most important bases for NagC binding to Box elements are the G or C bases at the -11 and +11 locations, and two conserved T bases at positions -5 and -6 [40]. Positions +4 thru +10 are AT rich, and the adenine insertion occurs in this region. If this insertion is viewed as shifting the BoxG1 sequence to the left, then the T at position -5 is changed to a C (Figure 3). Alternatively, if the extra adenine is viewed as shifting the sequence to the right, then the near-consensus G at position +11 is replaced by a T. In either case, it seems likely that the insertion reduces binding of the NagC activator to BoxG1, thereby reducing *glmUS *expression from the P1 promoter during growth on glucose.

To evaluate how this mutation affected *glmUS *expression, we examined data from a previous experiment [12] that measured, with four-fold replication, genomic expression profiles for the ancestor and for evolved clones from generations 2000, 10,000 and 20,000 in the same population that we used to isolate the BoxG1^8A ^mutation. As shown below, the BoxG1^8A ^mutation arose prior to generation 2000. The expression profiles were obtained for cells that were growing exponentially in the same glucose medium and other conditions of the long-term experiment. In all 24 comparisons between ancestral and evolved clones (4 replicates × 3 generations × 2 genes), the standardized expression (adjusted for total mRNA) was lower in the evolved clone, a pattern that is extremely unlikely by chance alone. The magnitude of the reduction was ~9% for both *glmU *and *glmS*, and at all three time points. These data therefore support the hypothesis that the BoxG1^8A ^mutation caused a subtle, but significant, reduction in the expression of *glmUS *during growth on glucose. Such a physiological change is also consistent with the reduced demand for peptidoglycan and lipopolysaccharide, relative to other cell constituents, in larger cells based on the geometry of surface area to cell volume.

### Fitness effects of the BoxG1^8A ^mutation in different media

The BoxG1^8A ^mutation is clearly beneficial in glucose, where the mutation evidently reduces binding of NagC to the BoxG1 site and thereby reduces expression of the *glmUS *operon. To examine further this allele's physiological effects, we performed competitions in the same minimal medium but replacing glucose with other substrates chosen for their relationships to *glmUS *and NagC.

First, the BoxG1^8A ^mutation has no significant effect during competition for UDP-GlcNAc, which is the end product of GlmS and GlmU activities (Figure 2). The fitness of TC640, which contains only the BoxG1^8A ^mutation on the ancestral background, relative to the ancestor in medium containing UDP-GlcNAc is 0.99 ± 0.03 (95% confidence interval). The absence of any effect is not surprising because UDP-GlcNAc is biochemically downstream of the *glmUS *operon.

Second, the BoxG1^8A ^allele is significantly advantageous in GlcN6P, which is a product of GlmS and is converted to GlcN1P before being a substrate for GlmU (Figure 2). The fitness of TC640 relative to the ancestor in medium containing GlcN6P is 1.15 ± 0.09 (95% confidence interval). At first glance, this advantage might suggest that expression of *glmUS *is increased in this case. Alternatively, GlcN6P can be converted by the *nagB*-encoded deaminase to F6P [41], which can be catabolised through the glycolytic pathway. GlmS acts in the opposing direction to covert Fru6P to GlcN6P for the production of cell-wall constituents. Hence, a reduction in *glmUS *expression caused by the BoxG1^8A ^allele may provide an advantage in GlcN6P by better balancing metabolic flux between catabolic and anabolic processes. We note, however, that this hypothesis is further complicated by the observation that GlcN6P interacts directly with NagC to reduce its affinity for at least some binding sites [42]. Third, and consistent with this second hypothesis for the advantage of BoxG1^8A ^in GlcN6P, the BoxG1^8A ^allele confers a significant benefit in competition for fructose, which is also catabolized to Fru6P. The fitness of TC640 relative to the ancestor in medium containing fructose as the sole carbon source is 1.06 ± 0.03 (95% confidence interval).

Fourth, the BoxG1^8A ^mutation confers a small but significant disadvantage during competition for glucosamine (GlcN). The fitness of TC640 relative to the ancestor in medium with GlcN as the sole source of carbon is 0.95 ± 0.02 (95% confidence interval). GlcN enters the cell via the PTS, which converts it to GlcN-6-phosphate [41]. By contrast, GlcN6P enters the cell via a permease that functions for many phosphorylated sugars [41]. The finding that the BoxG1^8A ^allele is detrimental in GlcN while beneficial in GlcN6P suggests some effect on transport, presumably mediated by NagC and its interaction with the BoxG1 site. GlcN enters the cell via two different PTS enzymes: II^Glc ^encoded by *ptsG*, and II^Man ^encoded by *manY *(*ptsM*) [41]. The latter route is the main one [36]. NagC is a repressor of the *manXYZ *operon as well as of the *nagE-BACD *operon [36]. Thus, NagC represses its own synthesis, although it also has constitutive promoters that stabilize its expression [36]. Given these interactions, it is unclear exactly how the feedback would impose a disadvantage to the BoxG1^8A ^allele in medium containing GlcN, but clearly the potential exists for complex regulatory effects. One possibility is that the reduced binding of NagC to the mutant BoxG1 site leaves more NagC to bind to the *manXYZ *operator, thereby slightly repressing it and impeding GlcN transport and catabolism. The growth rate of *E. coli *in GlcN has been shown to be limited by transport, and so any decrease in *manY *expression might reduce fitness [43]. Fifth, and consistent with this last hypothesis, the BoxG1^8A ^allele is slightly disadvantageous in competition for mannose, which is also taken up through the II^Man ^PTS system. The fitness of TC640 relative to the ancestor in mannose medium is 0.97 ± 0.02 (95% confidence interval).

We must emphasize that the possible explanations suggested above for the fitness effects of the BoxG1^8A ^allele in various media are hypotheses, not conclusions. We would welcome further biochemical and molecular studies of this mutation, and others like it, that might confirm or refute these hypotheses.

### Substitution dynamics of the beneficial mutation

To better understand the evolutionary dynamics of the BoxG1^8A ^mutation, we sought to determine when it appeared in the evolving population and the time course of its substitution. The clone from which REL10247 was derived was isolated at generation 10,000. Preliminary sequencing of the BoxG1 region from samples taken at earlier times indicated that the mutation was spreading between 500 and 1500 generations. We therefore chose many clones at random from the population samples that had been stored at generations 500, 1000, and 1500 generations, and we sequenced the BoxG1 region in each. None of 51 clones from generation 500 had the BoxG1^8A ^mutation, whereas 14/31 (45%) clones carried it at generation 1000, and 29/30 (97%) clones had this mutation at generation 1500.

In an evolving asexual population, the actual rate of spread of a beneficial mutation is often slower than expected from its selective advantage. This discrepancy arises from a phenomenon called clonal interference, whereby a clone with a particular beneficial mutation must compete with clones bearing other beneficial mutations [27,44-46]. The decelerating effect of this interference on the mutation that ultimately prevails can be quite pronounced, because those other clones that reach high frequency tend to be strong competitors. To examine the possible effects of clonal interference on the substitution dynamics of the BoxG1^8A ^mutation, we performed pairwise competitions between ten clones that were isolated at generation 1000 from this population, five carrying the beneficial BoxG1^8A ^allele and five with the ancestral BoxG1^7A ^allele. From each clone, we then isolated a spontaneous Ara^+ ^mutant; such mutations have been shown to be effectively neutral in the glucose-limited environment used in the long-term evolution experiment, but they allowed us to distinguish the competitors by plating on TA indicator plates. Each BoxG1^8A ^clone was competed with each BoxG1^7A ^clone, with 2-fold replication corresponding to the reciprocal Ara marker states, for a total of 50 competitions. The grand mean fitness of BoxG1^8A ^clones relative to BoxG1^7A ^clones was only 1.0075, which indicates an advantage of less than 1%. Although the BoxG1^8A ^clones were slightly more fit, this advantage is far smaller than the ~5% advantage measured relative to the ancestor. These data imply strong interference from competing clones that carry other beneficial mutations with the spread of the BoxG1^8A ^mutation.

### Sequencing *glmUS *promoter region in other evolved lines

Having discovered the BoxG1^8A ^mutation in our focal population and shown that it was beneficial, this element and the entire *glmUS *promoter region became a logical candidate region for sequencing in the other 11 populations founded with the same ancestral strain and propagated in the same environment. We sequenced the *glmUS *promoter region in clones sampled at 10,000 and 20,000 generations from all of the populations. The focal population retained the BoxG1^8A ^allele at generation 20,000. However, all 11 other populations still had the ancestral sequence at both time points. Evidently, only one population substituted this particular mutation, although all 12 populations achieved substantial fitness gains during the experiment [17,18].

## Discussion

### Chance and necessity

We began this paper by noting the tension between the roles of chance and necessity in evolution. We sought to address this issue by finding a beneficial mutation in one long-term experimental population of *E. coli*, without relying on any candidate loci, and then sequencing the region in other replicate populations to determine whether they had substituted the same or similar mutations. We found a beneficial mutation in the BoxG1 protein-binding site located near the P1 promoter of the *glmUS *operon. The mutation was a 1-bp insertion of an adenine in a tract of seven existing adenines. The mutation spread in its source population between generation 500, when it was rare or absent, and generation 1500, when it was present in almost every cell. The mutation persisted through 20,000 generations. It conferred a substantial fitness advantage, on the order of 5%, when transduced into the ancestor.

We also sequenced the region upstream of *glmUS *in 11 replicate populations. These other populations were founded by the same ancestor, and were propagated for 20,000 generations in the same environment, as the population that had evolved the BoxG1^8A ^beneficial mutation. Moreover, these replicate populations all had substantial fitness gains of similar magnitude to the gain measured in the population that substituted the BoxG1^8A ^allele [17,18]. However, none of 11 other populations evolved a change in BoxG1 nor anywhere else in the region upstream of the *glmUS *operon. Several other adaptive changes have occurred in only one of the replicate populations [47,48]. These findings illustrate the unpredictability of evolution, not merely at the level of random drift of neutral mutations, but instead with respect to adaptation. In striking contrast to these unique events, however, many changes in the evolving populations involve repeated beneficial mutations in the same genes [11-13,21-23,28]. Taken together, these cases nicely demonstrate that adaptation by natural selection can be both predictable and unpredictable in the same experiment.

It is interesting to consider two alternative hypotheses for why a BoxG1 mutation was substituted in only one of 12 replicate populations, despite its substantial advantage in the ancestral genetic background. According to one hypothesis, there have not been enough cell generations in each population for the same mutation to have occurred in the other populations. According to the other hypothesis, different mutations were substituted in the replicate populations that rendered this particular mutation no longer advantageous. The former hypothesis captures the idea of chance in its simplest sense of individual stochastic events. The latter explanation adds historical contingency, such that the likelihood of a particular outcome is conditional on whether some other event has already occurred [47].

The following quantitative considerations argue against the adequacy of the first and simpler hypothesis. The size of each population fluctuated between about 5 × 10^6 ^and 5 × 10^8 ^cells, owing to the daily dilution into fresh medium and re-growth. The effective population size with respect to new beneficial mutations escaping extinction by random drift is approximately equal to the minimum size multiplied by the number of generations between minimum and maximum sizes [16]: 5 × 10^6 ^× log_2 _100 ≈ 3 × 10^7 ^cells. The mutation rate in *E. coli *has been estimated to be 5 × 10^-10 ^per bp by Drake [49], although the substitution rate of synonymous mutations in the long-term experiment yields a somewhat lower estimate of 1.5 × 10^-10 ^[26,28]. Even using this lower rate estimate and the effective population size, and with three alternative base-pairings at each site, one expects that during 20,000 generations the average mutation has occurred (3 × 10^7^) × (1.5 × 10^-10^/3) × (2 × 10^4^) = 30 times. The 1-bp insertion of an adenine into an existing tract of seven adenines in the BoxG1 element is likely to have occurred more often, because homopolymeric tracts are very prone to insertions and deletions resulting from strand slippage during replication [50,51]. Moreover, several of the long-term populations evolved defects in their DNA repair, causing much higher mutation rates [18,26,52]. Of course, many beneficial mutations are lost by random drift while they are rare. The probability of a beneficial mutation being lost by drift is approximately 1 - 2*s*, where *s *is its selective advantage [44,53]. Thus, a mutation that confers a 5% advantage, such as the one in BoxG1, is expected to be lost by drift ~90% of the time. Even so, one would expect such a mutation to have appeared and survived drift multiple times in each population. Yet, the BoxG1^8A ^mutation was substituted in only 1 of the 12 evolving populations.

How can we explain its absence from the other populations? The best explanation, in our view, relies on the effects of clonal interference and epistasis, which together give rise to the historically contingent form of chance. In an asexual population, such as in the long-term experiment, two clones that acquire different beneficial mutations will compete with one another, and only one can ultimately prevail. Owing to this clonal interference, many beneficial mutations that escape drift nonetheless will be lost in competition with superior mutations [44,45]. We showed that clonal interference impeded the rate of spread of the BoxG1^8A ^mutation even in the population in which it was substituted. Although clonal interference might explain the failure to observe this mutation in the other populations, we doubt that it alone suffices. The likelihood that a given beneficial mutation will be eliminated by clonal interference depends on its advantage relative to other beneficial mutations still being generated in the evolving population [44]. Early in the long-term evolution experiment, a number of mutations with benefits on the order of 10% spread through all of the populations, and these could have interfered with the survival of the BoxG1^8A ^mutation [16,17]. However, the rate of fitness improvement declined sharply after the first 2000 generations [17,18,54]. Therefore, later appearances of the BoxG1^8A ^mutation would not have encountered many superior beneficial mutations, assuming that the BoxG1^8A ^mutation itself would still have conferred a ~5% advantage in these evolved genetic backgrounds. Yet, these BoxG1^8A ^mutations were not seen, even much later, in the other 11 populations. This absence suggests, therefore, that mutations in other genes were substituted that rendered the BoxG1^8A ^mutation less advantageous, neutral, or even disadvantageous. That is, some other mutations may interact epistatically with the BoxG1^8A ^mutation, such that their combined benefits are less than expected from their individual effects. After one of these other mutations had been substituted in a population, the likelihood of a BoxG1^8A ^mutation being substituted would therefore be reduced or eliminated.

### Mode of action of the BoxG1 beneficial mutation

Our experiments demonstrate that the BoxG1^8A ^allele is beneficial in the ancestral background in the glucose-limited medium. The mutation is located in the upstream regulatory region of the *glmUS *operon, which encodes two proteins involved in the synthesis of peptidoglycan and other cell-wall components. The BoxG1 motif has been shown previously to be a NagC protein-binding site that affects the transcription of nearby genes [31].

Individual *E. coli *cells become larger as they grow faster [55-57], and the glucose concentration used in the long-term experiment permitted growth at a rate that was extravagant compared with the natural environments in which this species evolved for millions of years prior to our evolution experiment [58]. Given the geometric fact that the ratio of surface area to volume is reduced in larger cells, it may have been beneficial for cells to produce reduced amounts of cell-wall constituents in this experiment. And given the role of GlmS and GlmU in producing these components, one might expect the BoxG1^8A ^allele to be associated with reduced expression of this operon. Indeed, a comparison of the BoxG1^8A ^sequence with the consensus Box sequence suggests reduced binding by the NagC activator of *glmUS *expression. Moreover, whole-genome expression profiles showed that evolved clones from this population, when growing exponentially in the same conditions of the long-term experiment, had consistently reduced mRNA levels for both *glmU *and *glmS *relative to the ancestor. Thus, the BoxG1^8A ^allele in the long-term evolution experiment evidently leads to reduced expression of the *glmUS *operon, which might provide a better balance between the synthesis of cell-wall and other cellular components.

As an added benefit, the end product of GlmS and GlmU catalysis is UDP-GlcNAc, a precursor to peptidoglycan (as well as other cell-wall components). The next step in peptidoglycan production is the transfer of enolpyruvate from phosphoenolpyruvate (PEP) to UDP-GlcNAc [32,33]. PEP is important not only as a precursor metabolite but also as the source of the high-energy phosphate that drives the phosphotransferase system (PTS), by which glucose is actively transported into the cell [59]. All else equal, reductions in the expression of *glmUS *and synthesis of UDP-GlcNAc should leave more PEP available for driving the PTS and thereby acquiring glucose, the sole available source of carbon and energy in the long-term experiment. Consistent with the importance of PEP in this experiment, the population that evolved the BoxG1^8A ^allele later also substituted an IS*150 *insertion into *pykF*, which encodes an enzyme catalyzing the conversion of PEP to pyruvate [25]. This insertion presumably disrupts PykF function, causing the accumulation of PEP that could be used to drive the PTS-mediated acquisition of glucose. In addition to its advantage in medium containing glucose, the BoxG1^8A ^mutation has significant effects on competitiveness in media containing fructose, GlcN6P, GlcN, and mannose, possibly through NagC-mediated effects on sugar transport and catabolism genes.

All 12 populations in the long-term experiment evolved larger cell size, but only the Ara-1 population substituted a mutation anywhere in the upstream regulatory region of the *glmUS *operon. However, several populations, including Ara-1, substituted mutations either in the *pbpA *gene or near the promoter of *pbpA-rodA*, another operon involved in peptidoglycan production and cell-wall elongation [13,19,21]. Moreover, mutations in two additional genes involved in cell-wall synthesis, *mrdB *and *mreB*, rose to intermediate frequency in the Ara-1 population in the first 2000 generations, before they were eliminated, presumably by clonal interference [27]. Thus, although the substitution in *glmUS *is unique to the Ara-1 population, beneficial mutations in other genes encoding related functions arose in many of the replicate populations.

In contrast to our explanation, Graña and Acerenza [20] proposed a model in which the parallel increases in cell size and growth rate of these long-term populations were both consequences of diverting intracellular resources from unnecessary physiological functions to those that enhanced performance in the glucose-only regime. According to the model, beneficial mutations need not have a direct role in cell-wall synthesis in order to impact cell size. However, their model and our explanation are not mutually exclusive. In fact, the evolution of faster growing and larger cells caused by loss or repression of unneeded functions should intensify the selection to reduce the relative production of cell-wall components, owing to the lower surface-to-volume ratio.

## Conclusions

In this study, we sought to discover and characterize one of the beneficial mutations responsible for the evolutionary adaptation in one of 12 *E. coli *populations grown for 20,000 generations in glucose-supplemented minimal medium. We then wanted to see whether the same or a similar mutation had occurred in the other populations, in order to examine the roles of chance and necessity in adaptive evolution. Our strategy employed transposon-tagging an evolved clone's genome followed by transduction into the ancestor, screening for increased cell size and fitness, co-transduction analysis, and sequencing the relevant region in the focal and other populations. Our main conclusions are as follows:

1) We found a 1-bp insertion in the BoxG1 region located in one of two operators of *glmUS*, an operon involved in synthesizing peptidoglycan and other components of the cell wall. The BoxG1 element has been shown previously to bind NagC, which activates this promoter during growth on glucose.

2) This mutation spread through the focal population between generations 500 and 1500. When transduced into the ancestral background, the BoxG1^8A ^allele increased competitive fitness by about 5%.

3) Expression profiles of ancestral and evolved clones from the focal population show a reduction of almost 10% in *glmUS *expression associated with the substitution of the BoxG1^8A ^allele. The insertion of the extra base-pair into the BoxG1 site apparently reduces NagC binding and thereby the activity of the P1 promoter during growth on glucose.

4) Faster growing cells are larger and, consequently, have reduced surface-to-volume ratios; therefore, they have proportionately less need for cell-wall components. The BoxG1 mutation and reduced expression of *glmUS *may thus confer an advantage by reducing excess synthesis of these components. Also, the end-product of GlmS and GlmU catalysis is UDP-GlcNAc, a precursor to peptidoglycan. The next step in the production of peptidoglycan consumes PEP, which would otherwise be available to drive PTS-mediated glucose transport.

5) Despite the large fitness gain conferred by the BoxG1^8A ^allele in the environment in which it arose, no similar mutations were found upstream of the *glmUS *operon in any of the other 11 replicate populations, even after 20,000 generations. A possible explanation is that those populations substituted mutations at other loci that interact epistatically with the *glmUS *operon, so that mutations in the BoxG1 region would no longer confer a substantial benefit. In fact, mutations in another operon involved in cell-wall synthesis arose in a number of replicate populations, including the focal population with the *glmUS *mutation. In any case, the unique substitution at the BoxG1 site illustrates the unpredictability of adaptive evolution, whereas parallel substitutions at other loci in these same populations illustrate its predictability.

## Methods

### Bacterial strains and culture conditions

The bacteria used in this study are part of a long-term evolution experiment that has been described in detail elsewhere [16-18]. Briefly, 12 populations of *Escherichia coli *B were started, six from each of two genetically marked variants of the ancestor. The populations were propagated by 1:100 daily serial transfer in Davis minimal medium supplemented with glucose at 25 μg per ml (DM25) at 37°C for 20,000 generations (3000 days). The two ancestral variants, REL606 and REL607, are phenotypically Ara^- ^and Ara^+^, respectively. These variants are selectively neutral in DM25 medium, but Ara^- ^and Ara^+ ^genotypes can be distinguished by their red and white colonies, respectively, on tetrazolium-arabinose (TA) indicator agar. At 500-generation intervals, samples from each population were stored at -80°C.

The source of the beneficial BoxG1^8A ^mutation that is the focus of this paper is a clone, REL4548, that was isolated from population Ara-1 at generation 10,000. This population retained functional DNA repair throughout the first 20,000 generations of the evolution experiment, whereas several other populations became hypermutable as a consequence of mutations that disrupted repair functions [18,52]. The Ara-1 population also subsequently evolved a mutator phenotype, but did so only after the 20,000 generations examined in this study [27,28]. REL10247 is the designation of a P1-transductant clone that was derived using REL4548 as the donor and REL606 as the recipient. The REL10247 genome has a mini-transposon bearing a tetracycline resistance (Tet^r^) marker inserted 20 bp downstream from the last codon in the *glmUS *operon; and a 1-bp adenine insertion in a tract of seven pre-existing adenines in the BoxG1 site of the *glmUS *promoter region. It has no other mutational differences from the ancestor within ~6,000 bp on either side of the mini-transposon. However, it has a deletion of the *rbs *operon [11] that begins ~18,000 bp from the BoxG1 site (in the direction opposite from the mini-transposon), which was co-transduced from REL4548. We describe later the construction of another strain, TC640, that carries the evolved BoxG1^8A ^allele but is otherwise isogenic to the ancestor.

When sequencing BoxG1 and the rest of the region upstream of the *glmUS *operon, we chose clones at random from the frozen samples of the Ara-1 population taken at various time points, as well as from samples of the other 11 populations at generations 10,000 and 20,000.

Strain JM109 and plasmid pUC19 were obtained from New England Biolabs and used in certain genetic manipulations described below.

### Mini-transposon tagging

The protocol that we used to produce a library of clones carrying mini-transposons and associated markers follows the one developed by Kleckner et al. [60]. Phage λNK1323 was used as the delivery vector for inserting mini-Tn*10 *transposons that confer tetracycline resistance into REL4548. We made 1296 independent insertion mutants. These mutants were used to inoculate individual 2-ml cultures of LBC supplemented with 15 μg/ml Tet, which were grown overnight at 37°C. This number of insertion mutants is such that, in a genome of ~4.6 × 10^6 ^bp [30], a marker should occur, on average, every ~3600 bp. The 1296 cultures were then mixed in equal volume to provide a proportional representation of each marked clone. Glycerol was added to 16%, and this mixture, designated MIX-1, was frozen at -80°C for later use in producing the P1-transducing lysate.

### P1-mediated transduction and co-transduction

Following a standard protocol [61], phage P1 (ATCC25404-B1) was grown on MIX-1 to produce a lysate stock (LYS-1) that was then used to transduce the ancestral strain REL606. An aliquot of the transduced culture was plated on LB agar containing 15 μg/ml Tet to estimate the total number of Tet^r ^transductants. The remaining culture was added to 200 ml LB supplemented with 15 μg/ml Tet, allowed to grow overnight, then frozen at -80°C in 16% glycerol. This frozen culture, designated MIX-2, thus contained a diverse mix of genotypes. These genotypes consist of randomly-tagged DNA from the evolved clone REL4548 bearing a mini-transposon along with any physically linked DNA sequences, inserted into the ancestral REL606 background. P1 phage transfers only ~2% of the *E. coli *genome between donor and recipient strains. Another clone sampled from the same population and generation has a total of 28 mutations distributed throughout its sequenced genome [28], and so one would expect that most transductants in the MIX-2 culture carry no more than 1 or 2 mutations.

We also performed a second round of transduction to estimate the physical distance between the beneficial mutation and the mini-transposon marker, based on their co-transduction. A P1-transducing lysate was prepared from REL10247 using the same methods as above. This lysate, designated LYS-2, was used to transduce the ancestral strain REL606. A random sample of the Tet^r ^transductants was screened for cell size, and some transductants were also used in fitness assays.

### Enrichment procedure to find beneficial transductant

To facilitate finding a clone that carried a beneficial mutation linked to a transposon-encoded marker, we serially propagated the MIX-2 mixture of transductants in the same DM25 medium and other culture conditions as used in the long-term evolution experiment. This procedure was performed for 7 daily cycles, corresponding to ~47 cell generations. Glycerol was then added to the resulting culture, designated MIX-3, which was stored at -80°C.

### Construction of isogenic mutant strain by gene gorging

To determine the phenotypic effects caused specifically by the BoxG1^8A ^mutation, we used the gene-gorging method [34] to move this mutation into the ancestral genome, producing a new strain designated TC640. Primers were designed to amplify a 1280-bp product centered on the BoxG1^8A ^mutation, one of which also contained the I-*Sce*I homing endonuclease recognition site required for the gene gorging protocol. These primers were used to amplify a product from REL4548, the 10,000-generation clone from which REL10247 was derived. This product was cloned into pCR2.1 to make a gene-gorging mutagenesis plasmid. Subsequent manipulations followed the previous protocol [34], except LB was used instead of rich defined medium. Successful transfer of the BoxG1^8A ^mutation was confirmed by direct sequencing. To ensure that no other mutations were inadvertently introduced during construction of TC640, we then also replaced the BoxG1^8A ^mutation with the ancestral operator sequence. The fitness of this reverted strain in glucose medium was indistinguishable from the ancestor (data not shown), which strongly implies that no secondary mutations affecting fitness were present in the intermediate strain, TC640.

### Screening for cell size

Previous research has shown a strong, positive correlation between cell volume and relative fitness in the evolving population studied here [17,19]. Moreover, cell size can be measured more easily and precisely than relative fitness. Therefore, we chose to focus on clones from MIX-3 that produced cells with significantly larger volumes than those produced by the ancestor REL606. Clones from MIX-3 were isolated on LB plates containing 15 μg/ml Tet, then propagated for two daily transfers in DM25 at 37°C. Average cell volumes were measured using a Coulter particle counter (model ZM and channelyzer model 256). The ancestor produces an average cell size of 0.37-0.39 fl, depending on the experiment, whereas the clones we chose for further study had average cell sizes of at least 0.44 fl. For the co-transduction analysis using REL10247 as donor and the ancestor as recipient, we scored Tet^r ^clones with average cell volumes ≥0.44 fl as having the beneficial allele, and those with averages ≤0.39 fl as having the ancestral allele; intermediate values were ignored when estimating the co-transduction frequency.

### Fitness assays

Competition experiments were performed to estimate the relative fitness of various clones. The procedures for the assays have been described in detail elsewhere [16]. In brief, two competing clones, one Ara^- ^and the other Ara^+^, are separately acclimated to the conditions in which they will compete, and then mixed at a 1:1 volumetric ratio in the competition environment. Samples are plated on TA agar immediately after mixing and again at the end of the competition experiment. The competitions ran for one complete serial-transfer cycle, which encompasses the same lag, growth, and stationary phases that occur in the long-term evolution experiment. Relative fitness is defined simply as the ratio of the net growth rates realized by the two strains during their competition [16].

Unless otherwise indicated, competitions involving particular clones of interest were performed against REL607, the Ara^+ ^ancestral strain. The competitions performed to study clonal interference used pairs of evolved clones, with spontaneous Ara^+ ^mutants having been obtained for each clone (on minimal arabinose agar) and the competition experiments balanced with respect to the Ara marker. Also unless otherwise noted, experiments were performed in DM25 medium, in which glucose at 25 μg/ml is the only usable source of carbon. To investigate the physiological effects of the BoxG1^8A ^mutation, additional competitions of TC640 against REL607 were performed in the same minimal medium except replacing the glucose with fructose, GlcN, GlcN6P, mannose, or UDP-GlcNAc. For UDP-GlcNAc only, the amount of resource was reduced to 8.75 μg/ml to equalize the available carbon.

### Southern blots

Plasmid pNKtet [60] was digested with *Bgl*I and *Xba*I, and the fragment containing the tetracycline-resistance gene was used as a probe. Genomic DNA of the clone designated REL10247 was isolated from an overnight LB culture using the Qiagen Genomic-tip system. A number of restriction enzymes were then used to digest this genomic DNA, including *Ava*I, *BamH*I, *Pvu*I, and *Pvu*II. Five μg of DNA were cut with 40 units of each restriction enzyme. The samples were run on a 0.5% agarose TBE gel, and then blotted onto a positively charged nylon membrane (Boehringer Mannheim) using a vacuum. Southern hybridization was performed using the DIG DNA Labelling and Detection Kit (Boehringer Mannheim) and the manufacturer's recommendations. The lane containing the *Ava*I-digested genomic DNA produced a band of ~5500 bp containing the entire functional tetracycline-resistance marker [60] as well as flanking DNA. The size of this fragment made it suitable to clone it and then sequence the genomic DNA flanking the mini-transposon.

### Mapping and sequencing

To map the chromosomal location of the mini-transposon inserted in the REL10247 genome, the ~5500 bp *Ava*I fragment was excised, purified, cloned into pUC19, and used to transform JM109. Prior to ligation, the *Ava*I-digested vector was treated with alkaline phosphatase (Gibco BRL) following the manufacturer's recommendations for 5' overhangs. The ligation was performed at 16°C for 16 h after a 5' treatment at 45°C. Following transformation of JM109, a Tet^r ^clone was isolated on an LB plate containing 15 μg/ml Tet. The DNA insert was sequenced using M13/pUC forward and reverse primers. A BLAST search was used to locate the site of the transposon insertion. The RL100 primer (5'-CGGATCCGATCATATGACAAGATGTGTA-3') was used specifically for the junction between the transposon and the *E. coli *genome.

### Primers and PCR

We sequenced regions around the mini-transposon insertion via chromosome walking, in order to find the beneficial mutation that was co-transduced with the insertion in REL10247. To that end, genomic DNA was isolated from REL606 and REL10247. PCRs were then used to generate four partially overlapping fragments, ranging in size from ~3400 to ~4100 bp, which were purified using an Ultrafree-MC filter (Millipore) or an Elu-Quik DNA Purification Kit (Schleicher & Schuell). The PCR fragments were sequenced on each end, and new sequencing primers were designed at the 3' end of the prior sequencing run. Sequencing continued until both ends overlapped. The *glmUS *promoter region was sequenced on both strands using primer RL36 (positive strand: 5'-ATTTTCTGCAGACAAAAGGCGTGAC-3') and RL51 (negative strand: 5'-GTTCTCGGTGGTGCGGATAACAAT-3').

Some of our experiments involved sequencing the *glmUS *promoter region from many clones. These PCRs used primers RL32 (5'-GTTCTGGCCGACACCGCAAT-3') and RL35 (5'-GCAGTTTTTCAGCCTGTTCGGACT-3').

Sequence data were managed using the DNASTAR software (Madison, Wisconsin).

## Abbreviations

CI: confidence interval; Fru: fructose; GlcN: glucosamine; IS: insertion sequence; LPS: lipopolysaccharide; PEP: phosphoenolpyruvate; PTS: phosphotransferase system; Tet^R^: tetracycline resistant; UDP-GlcNAc: uracil-diphosphate-N-acetyl-glucosamine.

## Authors' contributions

MTS and TC performed the genetic and competition experiments reported here. REL was responsible for the overall direction of these experiments as well as the long-term evolution experiment. All authors contributed substantially to writing this paper, and approved the final manuscript.

## References

[B1] Monod J Chance and Necessity 1971 New York, Knopf

[B2] Sniegowski PD Lenski RE Mutation and adaptation: the directed mutation controversy in evolutionary perspective Ann Rev Ecol Syst 1995 26 553 578 10.1146/annurev.es.26.110195.003005

[B3] Wright S Evolution in Mendelian populations Genetics 1931 16 97 159 17246615 10.1093/genetics/16.2.97 PMC1201091

[B4] Gould SJ Wonderful Life: The Burgess Shale and the Nature of History 1989 New York, Norton

[B5] Bull JJ Badgett MR Wichman HA Huelsenbeck JP Hillis DM Gulati A Ho C Molineux IJ Exceptional convergent evolution in a virus Genetics 1997 147 1497 1507 9409816 10.1093/genetics/147.4.1497 PMC1208326

[B6] Cunningham CW Jeng K Husti J Badgett M Molineux IJ Hillis DM Bull JJ Parallel molecular evolution of deletions and nonsense mutations in bacteriophage T7 Mol Biol Evol 1997 14 113 116 9000760 10.1093/oxfordjournals.molbev.a025697

[B7] Treves DS Manning S Adams J Repeated evolution of an acetate cross-feeding polymorphism in long-term populations of *Escherichia coli* Mol Biol Evol 1998 15 789 797 9656481 10.1093/oxfordjournals.molbev.a025984

[B8] Wichman HA Badgett MR Scott LA Boulianne CM Bull JJ Different trajectories of parallel evolution during viral adaptation Science 1999 285 422 424 10.1126/science.285.5426.422 10411508

[B9] Notley-McRobb L Ferenci T Adaptive *mgl *regulatory mutations and genetic diversity evolving in glucose-limited *Escherichia coli *populations Environ Microbiol 1999 1 33 43 10.1046/j.1462-2920.1999.00002.x 11207716

[B10] Notley-McRobb L Ferenci T Experimental analysis of molecular events during mutational periodic selections in bacterial evolution Genetics 2000 156 1493 1501 11102352 10.1093/genetics/156.4.1493 PMC1461358

[B11] Cooper VS Schneider D Blot M Lenski RE Mechanisms causing rapid and parallel losses of ribose catabolism in evolving populations of *E. coli *B J Bacteriol 2001 183 2834 2841 10.1128/JB.183.9.2834-2841.2001 11292803 PMC99500

[B12] Cooper TF Rozen DE Lenski RE Parallel changes in gene expression after 20,000 generations of evolution in *E. coli* Proc Natl Acad Sci USA 2003 100 1072 1077 10.1073/pnas.0334340100 12538876 PMC298728

[B13] Woods R Schneider D Winkworth CL Riley MA Lenski RE Tests of parallel molecular evolution in a long-term experiment with *Escherichia coli* Proc Natl Acad Sci USA 2006 103 9107 9112 10.1073/pnas.0602917103 16751270 PMC1482574

[B14] Spiers AJ Kahn SG Bohannon J Travisano M Rainey PB Adaptive divergence in experimental populations of *Pseudomonas fluorescens*. I. Genetic and phenotypic bases of wrinkly spreader fitness Genetics 2002 161 33 46 12019221 10.1093/genetics/161.1.33 PMC1462107

[B15] Ostrowski EA Woods R Lenski RE The genetic basis of parallel and divergent phenotypic responses in evolving populations of *Escherichia coli* Proc R Soc Lond B 2008 275 277 284 10.1098/rspb.2007.1244 PMC2593719 18029306

[B16] Lenski RE Rose MR Simpson SC Tadler SC Long-term experimental evolution in *Escherichia coli*. I. Adaptation and divergence during 2,000 generations Am Nat 1991 138 1315 1341 10.1086/285289

[B17] Lenski RE Travisano M Dynamics of adaptation and diversification: a 10,000-generation experiment with bacterial populations Proc Natl Acad Sci USA 1994 91 6808 6814 10.1073/pnas.91.15.6808 8041701 PMC44287

[B18] Cooper VS Lenski RE The population genetics of ecological specialization in evolving *E. coli *populations Nature 2000 407 736 739 10.1038/35037572 11048718

[B19] Lenski RE Mongold JA Brown JH, West GB Cell size, shape, and fitness in evolving populations of bacteria Scaling in Biology 2000 Oxford, Oxford University Press 221 235

[B20] Graña M Acerenza L A model combining cell physiology and population genetics to explain *Escherichia coli *experimental evolution BMC Evol Biol 2001 1 12 10.1186/1471-2148-1-12 11782284 PMC64492

[B21] Philippe N Pelosi N Lenski RE Schneider D Evolution of penicillin-binding protein 2 concentration and cell shape during a long-term experiment with *Escherichia coli* J Bacteriol 2009 191 909 921 10.1128/JB.01419-08 19047356 PMC2632098

[B22] Crozat E Philippe N Lenski RE Geiselmann J Schneider D Long-term experimental evolution in *Escherichia coli*. XII. DNA topology as a key target of selection Genetics 2005 169 523 532 10.1534/genetics.104.035717 15489515 PMC1449116

[B23] Pelosi L Kühn L Guetta D Garin J Geiselmann J Lenski RE Schneider D Parallel changes in global protein profiles during long-term experimental evolution in *Escherichia coli* Genetics 2006 173 1851 1869 10.1534/genetics.105.049619 16702438 PMC1569701

[B24] Papadopoulos D Schneider D Meier-Eiss J Arber W Lenski RE Blot M Genomic evolution during a 10,000-generation experiment with bacteria Proc Natl Acad Sci USA 1999 96 3807 3812 10.1073/pnas.96.7.3807 10097119 PMC22376

[B25] Schneider D Duperchy E Coursange E Lenski RE Blot M Long-term experimental evolution in *Escherichia coli*. IX. Characterization of insertion sequence-mediated mutations and rearrangements Genetics 2000 156 477 488 11014799 10.1093/genetics/156.2.477 PMC1461276

[B26] Lenski RE Winkworth CL Riley MA Rates of DNA sequence evolution in experimental populations of *Escherichia coli *during 20,000 generations J Mol Evol 2003 56 498 508 10.1007/s00239-002-2423-0 12664169

[B27] Barrick JE Lenski RE Genome-wide mutational diversity in an evolving population of *Escherichia coli* Cold Spring Harbor Symposia on Quantitative Biology 2009 in press 19776167 10.1101/sqb.2009.74.018 PMC2890043

[B28] Barrick JE Yu D-S Yoon SH Jeong H Oh KT Schneider D Lenski RE Kim JF Genome evolution and adaptation in a long-term experiment with *Escherichia coli* Nature 2009 461 1243 1247 10.1038/nature08480 19838166

[B29] Low KB Neidhardt FC Genetic mapping Escherichia coli and Salmonella: Cellular and Molecular Biology 1996 Washington, DC, ASM Press 2511 2517

[B30] Jeong H Barbe V Lee CH Vallenet D Yu DS Choi S-H Couloux A Lee S-W Yoon SH Cattolico L Hur C-G Park H-S Ségurens B Kim SC Oh TK Lenski RE Studier FW Daegelen P Kim JF Genome sequences of *Escherichia coli *B strains REL606 and BL21(DE3) J Mol Biol 2009 394 644 652 10.1016/j.jmb.2009.09.052 19786035

[B31] Plumbridge J Co-ordinated regulation of amino sugar biosynthesis and degradation: the NagC repressor acts as both an activator and a repressor for the transcription of the *glmUS *operon and requires two separated NagC binding sites EMBO J 1995 14 3958 3965 7545108 10.1002/j.1460-2075.1995.tb00067.x PMC394474

[B32] Raetz CRH Neidhardt FC Bacterial lipopolysaccharides: a remarkable family of bioactive macroamphiphiles Escherichia coli and Salmonella: Cellular and Molecular Biology 1996 Washington, DC, ASM Press 1035 1063

[B33] van Heijenoort J Neidhardt FC Murein synthesis Escherichia coli and Salmonella: Cellular and Molecular Biology 1996 Washington, DC, ASM Press 1025 1034

[B34] Herring CD Glasner JD Blattner FR Gene replacement without selection: regulated suppression of amber mutations in *Escherichia coli* Gene 2003 331 153 163 10.1016/S0378-1119(03)00585-7 12853150

[B35] Plumbridge J Kolb A CAP and Nag repressor binding to the regulatory regions of the *nagE-B *and *manX *genes of *Escherichia coli* J Mol Biol 1991 217 661 679 10.1016/0022-2836(91)90524-A 1848637

[B36] Plumbridge J How to achieve constitutive expression of a gene within an inducible operon: the example of the *nagC *gene of *Escherichia coli* J Bacteriol 1996 178 2629 2636 8626331 10.1128/jb.178.9.2629-2636.1996 PMC177988

[B37] Plumbridge J Kolb A DNA loop formation between Nag repressor molecules bound to its two operator sites is necessary for repression of the *nag *regulon of *Escherichia coli *in vivo Mol Microbiol 1993 10 973 981 10.1111/j.1365-2958.1993.tb00969.x 7934873

[B38] Plumbridge J Kolb A Nag repressor-operator interactions: protein-DNA contacts cover more than two turns of the DNA helix J Mol Biol 1995 249 890 902 10.1006/jmbi.1995.0346 7791215

[B39] Plumbridge J Kolb A DNA bending and expression of the divergent *nagE-B *operons Nucleic Acids Res 1998 26 1254 1260 10.1093/nar/26.5.1254 9469834 PMC147383

[B40] El Quadi S Plumbridge J Switching control of expression of *ptsG *from the Mlc regulon to the NagC regulon J Bacteriol 2008 190 4677 4686 10.1128/JB.00315-08 18469102 PMC2446801

[B41] Lin ECC Neidhardt FC Dissimilatory pathways for sugars, polyols, and carboxylates Escherichia coli and Salmonella: Cellular and Molecular Biology 1996 Washington, DC, ASM Press 307 342

[B42] Plumbridge J Repression and induction of the *nag *regulon of *Escherichia coli *K12: the roles of *nagC *and *nagA *in maintenance of the uninduced state Mol Microbiol 1991 5 2053 2062 10.1111/j.1365-2958.1991.tb00828.x 1766379

[B43] Alverez-Añorve LI Calcagno ML Plumbridge J Why does *Escherichia coli *grow more slowly on glucosamine than N-acetylglucosamine? Effects of enzyme levels and allosteric activation of GlcN6P deaminase (NagB) on growth rates J Bacteriol 2005 187 2974 2982 10.1128/JB.187.9.2974-2982.2005 15838023 PMC1082822

[B44] Gerrish PJ Lenski RE The fate of competing beneficial mutations in an asexual population Genetica 1998 102/103 127 144 10.1023/A:1017067816551 9720276

[B45] Shaver AC Dombrowski PG Sweeney JY Treis T Zappala RM Sniegowski PD Fitness evolution and the rise of mutator alleles in experimental *Escherichia coli *populations Genetics 2002 162 557 566 12399371 10.1093/genetics/162.2.557 PMC1462288

[B46] Cooper TF Recombination speeds adaptation by reducing competition between beneficial mutations in populations of *Escherichia coli* PLoS Biology 2007 5 e225 10.1371/journal.pbio.0050225 17713986 PMC1950772

[B47] Blount ZD Borland CZ Lenski RE Historical contingency and the evolution of a key innovation in an experimental population of *Escherichia coli* Proc Natl Acad Sci USA 2008 105 7899 7906 10.1073/pnas.0803151105 18524956 PMC2430337

[B48] Rozen DE Lenski RE Long-term experimental evolution in *Escherichia coli*. VIII. Dynamics of a balanced polymorphism Am Nat 2000 155 24 35 10.1086/303299 10657174

[B49] Drake JW A constant rate of spontaneous mutation in DNA-based microbes Proc Natl Acad Sci USA 1991 88 7160 7164 10.1073/pnas.88.16.7160 1831267 PMC52253

[B50] Weiser JN Love JM Moxon ER The molecular mechanism of phase variation of *H. influenzae *lipopolysaccharide Cell 1989 59 657 665 10.1016/0092-8674(89)90011-1 2479481

[B51] Moxon RE Rainey PB Nowak MA Lenski RE Adaptive evolution of highly mutable loci in pathogenic bacteria Curr Biol 1994 4 24 33 10.1016/S0960-9822(00)00005-1 7922307

[B52] Sniegowski PD Gerrish PJ Lenski RE Evolution of high mutation rates in experimental populations of *E. coli* Nature 1997 387 703 705 10.1038/42701 9192894

[B53] Haldane JBS A mathematical theory of natural and artificial selection. V. Selection and mutation Proc Camb Phil Soc 1927 23 838 844 10.1017/S0305004100015644

[B54] De Visser JAGM Lenski RE Long-term experimental evolution in *Escherichia coli*. XI. Rejection of non-transitive interactions as cause of declining rate of adaptation BMC Evo Biol 2002 2 19 10.1186/1471-2148-2-19 PMC134600 12443537

[B55] Åkerlund T Nordström K Bernander R Analysis of cell size and DNA content in exponentially growing and stationary-phase batch cultures of *Escherichia coli* J Bacteriol 1995 177 6791 6797 7592469 10.1128/jb.177.23.6791-6797.1995 PMC177544

[B56] Bremer H Dennis PP Neidhardt FC Modulation of chemical composition and other parameters of the cell by growth rate Escherichia coli and Salmonella: Cellular and Molecular Biology 1996 Washington, DC, ASM Press 1553 1569

[B57] Mongold JA Lenski RE Experimental rejection of a nonadaptive explanation for increased cell size in *Escherichia coli* J Bacteriol 1996 178 5333 5334 8752359 10.1128/jb.178.17.5333-5334.1996 PMC178338

[B58] Savageau MA *Escherichia coli *habitats, cell types, and molecular mechanisms of gene control Am Nat 1983 122 732 744 10.1086/284168

[B59] Postma PW Lengeler JW Jacobson GR Neidhardt FC Phosphoenolpyruvate: carbohydrate phosphotransferase systems Escherichia coli and Salmonella: Cellular and Molecular Biology 1996 Washington, DC, ASM Press 1149 1174

[B60] Kleckner N Bender J Gottesman S Uses of transposons with emphasis on Tn*10* Methods Enzymol 1991 204 139 180 full_text 1658561 10.1016/0076-6879(91)04009-d

[B61] Miller JH A Short Course in Bacterial Genetics 1992 Cold Spring Harbor, New York, Cold Spring Harbor Laboratory Press

